# Accuracy of the NICE traffic light system in children presenting to general practice: a retrospective cohort study

**DOI:** 10.3399/BJGP.2021.0633

**Published:** 2022-05-17

**Authors:** Amy Clark, Rebecca Cannings-John, Megan Blyth, Alastair D Hay, Christopher C Butler, Kathryn Hughes

**Affiliations:** School of Medicine, Cardiff University, Cardiff.; Centre for Trials Research, Cardiff University, Cardiff.; Division of Population Medicine, School of Medicine, Cardiff University, Cardiff.; Centre for Academic Primary Care, Bristol Medical School, University of Bristol, Bristol.; Nuffield Department of Primary Care Health Sciences, University of Oxford, Oxford.; PRIME Centre Wales, Division of Population Medicine, School of Medicine, Cardiff University, Cardiff.

**Keywords:** child health, clinical prediction rule, general practice, retrospective studies, primary health care, International Classification of Diseases

## Abstract

**Background:**

The National Institute for Health and Care Excellence (NICE) traffic light system was created to facilitate the assessment of unwell children in primary care. To the authors’ knowledge, no studies have validated this tool in UK general practice.

**Aim:**

To evaluate the accuracy of this system for detecting serious illness in children presenting to general practice.

**Design and setting:**

A retrospective diagnostic accuracy study was undertaken, using a cohort of acutely unwell children aged <5 years presenting to general practice in England and Wales.

**Method:**

The traffic light categories of 6703 children were linked with hospital data to identify admissions and diagnoses. The sensitivity and specificity of these categories were calculated against the reference standard: a hospital-diagnosed serious illness within 7 days of GP consultation, measured using *International Classification of Diseases, 10th Revision* codes.

**Results:**

In total, 2116 (31.6%) children were categorised as ‘red’; 4204 (62.7%) as ‘amber’; and 383 (5.7%) as ‘green’. There were 139 (2.1%) children who were admitted to hospital within 7 days of consultation, of whom 17 (12.2%; 0.3% overall) had a serious illness. The sensitivity of the red category (versus amber and green) was 58.8% (95% confidence interval [CI] = 32.9 to 81.6) and the specificity 68.5% (95% CI = 67.4 to 69.6). The sensitivity and specificity of red and amber combined (versus green) was 100% (95% CI = 80.5 to 100) and 5.7% (95% CI = 5.2 to 6.3), respectively.

**Conclusion:**

The NICE traffic light system did not accurately detect children admitted with a serious illness, nor those not seriously ill who could have been managed at home. This system is not suitable for use as a clinical tool in general practice. Further research is required to update or replace the system.

## INTRODUCTION

Children with acute illnesses are a common presentation in general practice, and can be a diagnostic challenge for clinicians.^1^^,^^2^ This is primarily because of their presentation at an early stage of illness, where the signs and symptoms of non-serious and serious illnesses can appear similar.^3^ The majority of acute illnesses are self-limiting viral infections, although a minority of children may have a serious illness such as pneumonia or meningitis.^4^ The estimated incidence of these illnesses in children presenting to general practice is 1% per year.^3^^,^^5^ Despite this, fear and anxiety in parents with an unwell child are common.^6^^,^^7^

In an effort to help primary care clinicians confidently assess unwell children, the National Institute for Health and Care Excellence (NICE) created the ‘traffic light’ system.^8^ This tool categorises children into ‘green’, ‘amber’, or ‘red’ depending on their consulting clinical features; corresponding to a low, intermediate, or high risk of serious illness, respectively.

According to the tool, children in the green category can be managed at home. Children in the amber category can either be referred to hospital for assessment or sent home with safety-net advice. Children in the red category should be referred urgently for assessment in hospital.

The recent relaxation of restrictions during the COVID-19 pandemic has led to a dramatic increase in the prevalence of respiratory illness in young children, with emergency departments experiencing high demand from children, many of whom are not seriously ill.^9^^,^^10^ This illustrates the importance of an accurate primary care tool to identify those needing secondary care assessment.

Validation of the traffic light system within general practice has been performed once previously in 2013, using a small Dutch cohort of 506 febrile children.^11^ Additional studies have evaluated this tool within emergency department settings.^4^^,^^11^^–^^13^ However, to the authors’ knowledge, no studies have evaluated the NICE traffic light system in UK general practice. The aim of this study was to evaluate the accuracy of the NICE traffic light system for predicting serious illness in acutely unwell children aged <5 years presenting to UK general practice.

## METHOD

The Standards for Reporting of Diagnostic Accuracy guidelines were followed in the reporting of this study.^14^

### Sample formation

This retrospective cohort study involved the secondary analysis of a dataset collected for the Diagnosis of Urinary Tract Infection in Young Children (DUTY) study.^15^ The DUTY study was a prospective cohort study of acutely unwell children aged <5 years in primary care, which evaluated the presenting signs and symptoms of urinary tract infections (UTIs). Children from 233 sites in England and Wales (general practices, walk-in centres, and emergency departments) were consecutively recruited between 2010 and 2012 if they were constitutionally unwell owing to any acute illness and/or presenting with a UTI symptom described by NICE. The eligibility criteria for recruitment into the DUTY study are presented in Supplementary Table S1. The study presented here only includes the children from the DUTY study who presented to general practice.

**Table:** How this fits in

The National Institute for Health and Care Excellence (NICE) traffic light system is widely used in general practice for the assessment of unwell children; however, the majority of previous studies validating this tool have been conducted in secondary care settings. To that authors’ knowledge, no studies have validated this tool within UK general practice. This study found that the traffic light system cannot accurately detect or exclude serious illness in children presenting to UK general practice with an acute illness. The conclusion reached was that it cannot be relied on by clinicians for the assessment of acutely unwell children and that it is unsuitable for use as a clinical decision tool.

### Data processing

The children’s clinical features at the time of their GP presentation were mapped retrospectively to equivalent variables within the traffic light system as part of a separate study.^16^ Children were categorised as red if they had at least one red feature; as amber if they had at least one amber feature (and no red); and as green if they had no amber or red features. Children with missing data, preventing traffic light categorisation, were excluded from the analyses.

Routinely collected hospital data in England and Wales were accessed to identify admissions; provided by Hospital Episode Statistics (collected by NHS Digital) and the Patient Episode Database for Wales, respectively. The data collected from children during the DUTY study were linked to hospital data using the Secure Anonymised Information Linkage (SAIL) databank.

### Outcome definitions

The primary outcome of interest was an unplanned hospital admission with a serious illness within 7 days of initial GP consultation.

A ‘hospital admission’ was defined as a spell in hospital under the care of a consultant; assessment in the emergency department was not recorded as an admission unless the treating team decided to admit them. To ensure a strong association between consulting clinical features and any subsequent hospital admission, a 7-day follow-up period was chosen.^4^^,^^12^^,^^17^^–^^19^

The definition used in this study of ‘serious illness’ was created by identifying the NICE definitions used during the creation of the traffic light system and exploring previous literature.^2^^,^^4^^,^^13^^,^^20^^–^^22^ The *International Classification of Diseases, 10th Revision* (ICD-10) codes for these serious illnesses were used to create a reference table (see Supplementary Table S2). The principal serious illnesses included: sepsis, pneumonia, meningitis, and UTI.

### Statistical analysis

To evaluate the test performance of the NICE traffic light system, sensitivity and specificity were calculated separately for two thresholds of test positivity: designation of red category and designation of red or amber category. This allowed comparison of test performance between ‘high-risk’ and ‘intermediate to high-risk’ populations. Further calculations included 95% confidence intervals (CIs) and positive and negative predictive values.

Two sensitivity analyses were conducted. The first assessed the ability of the traffic light system to detect children admitted to hospital (with or without a serious illness), to reduce the impact of incorrect diagnoses coding within the routine data. The second evaluated the traffic light system when applied to a cohort of febrile children, chosen because the traffic light system was created by NICE to assess febrile children specifically. The NICE definitions of fever were used to identify this subgroup: measured or perceived elevation of body temperature above the normal daily variation (≥38 °C) by a parent or clinician.^23^

Analyses were performed using IBM SPSS Statistics (version 26). To comply with SAIL regulations, data with frequencies <5 were suppressed and presented as ‘<5’ or rounded to the nearest five.

## RESULTS

**Figure 1.**
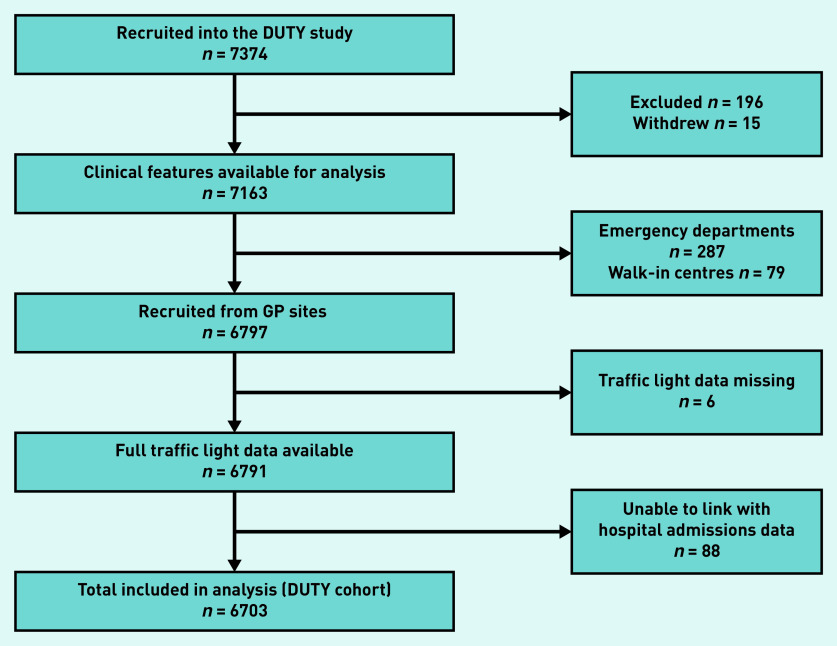
***Flow chart of children included in analysis.*** *^15^*

During the DUTY study period, 7374 children were recruited from primary care. Children presenting to care providers outside of general practice were excluded, in addition to those without available traffic light and hospital admissions data. Overall, 6703 (90.9%) children were eligible for inclusion in this study (Figure 1). This population of children presenting to general practice will be referred to as the ‘DUTY cohort’.

### Characteristics of the DUTY cohort

Nearly a quarter of the 6703 children attending general practice were aged between 1 and 2 (*n* = 1577, 23.5%, Table 1). The majority of children were categorised into amber (*n* = 4204, 62.7%), followed by red (*n* = 2116, 31.6%) and green (*n* = 383, 5.7%). The children in the green category were older, with a median age of 2.8 years (interquartile range [IQR] 1.4–3.8 years) compared with 2.0 years for children in both the amber (IQR 0.9–3.4 years) and red (IQR 1.1–3.3 years) categories, respectively (data not shown). The variable that triggered amber and red labels most commonly was ‘impaired social cues (moderate or severe)’, responsible for 97.0% and 61.2% of these categorisations, respectively. Abnormal vital signs including temperature, pulse rate, oxygen saturation, and respiratory rate contributed to an amber classification in 28.8% of children in the amber category and contributed to a red classification in 1.7% of the children in the red category. Further analyses of the traffic light categorisations are available as part of a separate article.^16^

**Table 1.** Characteristics of the DUTY children presenting to general practice by hospital admission status within 7 days

**Characteristic**	**DUTY cohort (*n* = 6703)**	**DUTY children not admitted to hospital (*n*= 6564)^a^**	**DUTY children admitted to hospital (*n* = 139)^a^**
**Age at recruitment, months,**			
***n* (%)**			
<3	198 (3.0)	191 (2.9)	7 (5.0)
3–5	409 (6.1)	395 (6.0)	14 (10.1)
6–11	1065 (15.9)	1041 (15.9)	24 (17.3)
12–23	1577 (23.5)	1541 (23.5)	36 (25.9)
24–35	1263 (18.8)	1248 (19.0)	15 (10.8)
36–47	1257 (18.8)	1227 (18.7)	30 (21.6)
≥48	934 (13.9)	921 (14.0)	13 (9.4)

**Age, years, median (IQR)**	2.1 (1.0–3.4)	2.1 (1.0–3.4)	1.6 (0.9–3.1)

**Sex,** ***n* (%)**			
Male	3282 (49.0)	3213 (48.9)	69 (49.6)
Female	3421 (51.0)	3351 (51.1)	70 (50.4)

**Traffic light category,** ***n* (%)**			
Green	383 (5.7)	378 (5.8)^b^	5 (3.4)^b^
Amber	4204 (62.7)	4154 (63.3)^b^	50 (34.5)^b^
Red	2116 (31.6)	2026 (30.9)^b^	90 (62.1)^b^

a *Within 7 days of the GP consultation.*

b *Figures have been rounded and adjusted to comply with SAIL ‘small data’ reporting regulations. DUTY = Diagnosis of Urinary Tract Infection in Young Children. SAIL = Secure Anonymised Information Linkage databank.*

### Hospital admissions

Linkage to hospital admissions data was achieved for 98.7% of children with available traffic light categories (*n* = 6703/6791). To ensure there was no selection bias between children who could not be linked to hospital data (*n* = 88) and children who could (*n* = 6703), a series of descriptive statistics were undertaken (see Supplementary Table S3). These confirmed no clinically significant differences in median age, duration of illness, or distribution of traffic light categories.

Within 7 days of presenting to general practice, 139 of 6703 children (2.1%) were admitted to hospital. The children admitted to hospital were younger than the children who were not admitted (Table 1). Additionally, more children were in the red category at initial presentation to general practice.

The median duration between general practice consultation and admission was 1 day (IQR 0–3 days), with just under half of patients admitted the same day (*n* = 57, 41.0%, data not shown). Children were most commonly discharged on the same day (*n* = 81, 58.3%) or the day after (*n* = 36, 25.9%). The median length of hospital stay was 0 days (IQR 0–1 day). The most common diagnosis in hospital was an unspecified viral infection (*n* = 20, 14.4%, Supplementary Table S4).

### Serious illnesses

**Figure 2.**
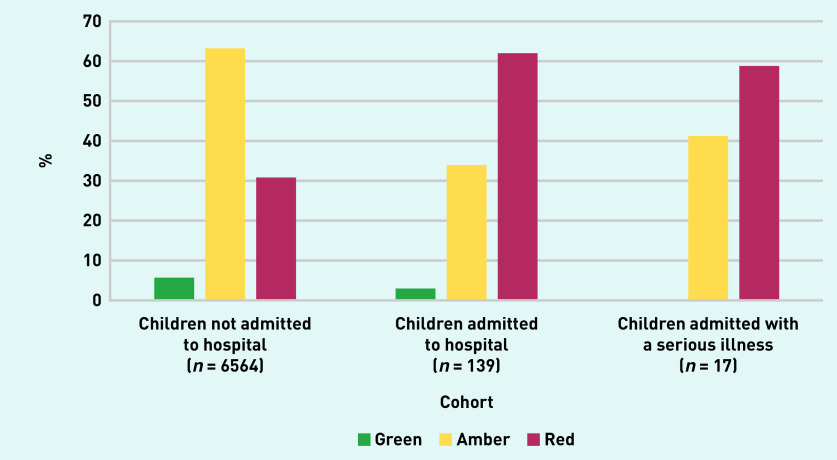
*Bar chart demonstrating the traffic light categories for children admitted and not admitted to hospital, and those with a serious illness. These figures have been rounded to the nearest five to comply with the ‘small data’ reporting requirements of the Secure Anonymised Information Linkage (SAIL) databank.*

The prevalence of serious illness in this cohort was 0.3% (*n* = 17/6703, 95% CI = 0.2 to 0.4). The majority of serious illnesses were cases of pneumonia (*n* = 8, 47.1%). No cases of sepsis or meningitis were reported (see Supplementary Table S4). Information on all diagnosed serious illnesses cannot be disclosed because of the small numbers. Of the children diagnosed with a serious illness, 10 (58.8%) were categorised as red at presentation to general practice (Figure 2). The median duration between general practice consultation and hospital admission in this group was 2 days (IQR 0–2 days) and the median length of hospital stay was 1 day (IQR 0–2 days, data not shown). Children were most commonly discharged on the same day (*n* = 7, 41.2%) or 2 days later (*n*<5).

### Performance of the NICE traffic light system

The red category had a sensitivity of 58.8% (95% CI = 32.9 to 81.6) and specificity of 68.5% (95% CI = 67.4 to 69.6) for detecting children admitted to hospital with a serious illness (Table 2). Combining the red and amber categories improved the sensitivity to 100% (95% CI = 80.5 to 100) but reduced the specificity to 5.7% (95% CI = 5.2 to 6.3).

The performance of the traffic light system for detecting any admission to hospital was also calculated (Table 3). The red category had a sensitivity of 61.9% (95% CI = 53.3 to 70.0) and specificity of 69.1% (95% CI = 67.9 to 70.2). Combining red and amber improved the sensitivity to 97.8% (95% CI = 93.8 to 99.6) but reduced specificity to 5.8% (95% CI = 5.2 to 6.4). The cross-tabulations used to calculate these measures of test performance are displayed in Supplementary Table S5.

**Table 2.** Prognostic performance of the NICE traffic light system for detecting serious illness

**Test performance for detecting children with a serious illness**	**Sensitivity, % (95% CI)**	**Specificity, % (95% CI)**	**Positive predictive value, %**	**Negative predictive value, %**
Red category only	58.8 (32.9 to 81.6)	68.5 (67.4 to 69.6)	0.5	99.8
Red or amber category	100 (80.5 to 100)	5.7 (5.2 to 6.3)	0.3	100

*NICE = National Institute for Health and Care Excellence.*

**Table 3.** Prognostic performance of the NICE traffic light system for detecting hospital admission

**Test performance for detecting children admitted to hospital**	**Sensitivity, % (95% CI)**	**Specificity, % (95% CI)**	**Positive predictive value, %**	**Negative predictive value, %**
Red category only	61.9 (53.3 to 70.0)	69.1 (67.9 to 70.2)	4.1	98.8
Red or amber category	97.8 (93.8 to 99.6)	5.8 (5.2 to 6.4)	2.2	99.2

*NICE = National Institute for Health and Care Excellence.*

### Sensitivity analysis for febrile children

In the sample in this study, 5032 children (75.1%) were febrile (data not shown). Of these, 120 (2.4%) were admitted to hospital and 16 (0.3%) were diagnosed with a serious illness. Therefore, 86.3% (*n* = 120/139) of children admitted to hospital and 94.1% (*n* = 16/17) of children with a serious illness were febrile at GP presentation. The traffic light system had improved sensitivity but poorer specificity for detecting serious illness when used in this population (Table 4).

**Table 4.** Prognostic performance of the NICE traffic light system for detecting febrile children with a serious illness

**Test performance for detecting serious illness in febrile children**	**Sensitivity, % (95% CI)**	**Specificity, % (95% CI)**	**Positive predictive value, %**	**Negative predictive value, %**
Red category only	62.5 (35.4 to 84.8)	64.0 (62.7 to 65.3)	0.6	99.8
Red or amber category	100 (79.4 to 100)	3.0 (2.5 to 3.5)	0.3	100

*NICE = National Institute for Health and Care Excellence.*

## DISCUSSION

### Summary

This study found that the prevalence of serious illness in 6703 acutely unwell young children presenting to UK general practice was 0.3% and that the NICE traffic light system categorised 31.6% of all children as red; 62.7% as amber; and 5.7% as green. Overall, 139 (2.1%) children were admitted within 7 days of their initial presentation. The traffic light tool had a sensitivity of 58.8% and specificity of 68.5% for the identification of children admitted to hospital with a serious illness, when comparing red with amber and green categories. Changing the threshold to include red and amber categories combined, compared with green, improved the sensitivity to 100% but worsened the specificity to 5.7%. The results were robust to detecting hospital admissions for any reason, or when applied to febrile children only.

### Strengths and limitations

To the best of the authors’ knowledge, this is the first study to validate the NICE traffic light system in UK general practice. A dataset of 6703 children was used, representing one of the largest and most detailed characterisations of clinical features among acutely unwell young children presenting to general practice in the UK, linked to hospital admissions data. Although the NICE guidelines and traffic light system were designed for febrile children, the inclusion of all children with an acute illness better represents the variety of children requiring assessment by GPs in clinical practice. Furthermore, the study included all illnesses defined by NICE as ‘serious’, which matches the illnesses that the traffic light system was designed to detect. It was also possible to provide a current prevalence estimate of serious illness in children presenting to general practice.

There are several limitations to this study. The prospective data were not collected specifically to answer this research question. Consequently, not all clinical features present in the traffic light system could be matched to the DUTY dataset during the assignment of traffic light categories.^16^ The variables that could not be mapped were mostly within the red category and principally involved neurological features such as neck stiffness, or orthopaedic signs such as limb swelling. If these features had been matched, potentially more children would have been in the red category, and the sensitivity of the traffic light system may have improved. However, most of the key constitutional features of the traffic light system were captured by the DUTY study, and 64% of data fields were mapped.^16^

The ‘serious illness’ reference standard used in this study was dependent on diagnostic codes within routinely collected hospital data; reference standards such as clinical, laboratory, and radiological data were not available to assess the evidence supporting a final diagnosis of a serious illness. It is possible that some children with a ‘serious illness’ were not identified in the study, due to incorrect coding of their diagnoses, thus underestimating the disease prevalence and tool sensitivity. However, the coding in this dataset represents the diagnoses recorded on discharge by a clinician with access to all investigations.

Finally, the sample in this study only included children who fulfilled the DUTY study eligibility criteria. Consequently, this cohort may not be entirely representative of all acutely unwell children within this age group. The criteria did not include children who were constitutionally well unless they had symptoms suggestive of a UTI. Children with focal illnesses or mild respiratory infections may have been excluded, and children with UTIs may have been overrepresented because of the aim of the DUTY study. However, the traffic light system was principally designed for constitutionally unwell children; therefore, the authors believe this cohort is adequately representative of this. There is a possibility that some of the children presenting to general practice were too ill to be recruited for the original study; however, the low disease prevalence is similar to previous estimates of serious illnesses presenting to general practice.^5^

### Comparison with existing literature

To the best of the authors’ knowledge, only one other study has validated the NICE traffic light system within general practice.^11^ This was a retrospective cohort study conducted by Verbakel *et al* in the Netherlands, using a sample of 506 febrile children aged from 3 months to 6 years. They reported a sensitivity of 100% and a specificity of 1% for the identification of serious bacterial infections, using the presence of any ‘red’ or ‘amber’ features as a positive test.^11^ These results are similar to the findings in the current study when red and amber categories were combined; however, it was not clear how Verbakel *et al* defined ‘serious infection’ — either as clinical judgement, hospital admission, or investigations performed in secondary care. Furthermore, information regarding the designation of traffic light categories was not provided.

Further studies have assessed the traffic light system in emergency department settings, reporting sensitivities between 85% and 99%, and specificities between 2% and 29% (using red or amber combined as a positive test).^4^^,^^11^^–^^13^ The results of the current study for red and amber combined were similar, with a particularly poor specificity. This may be because of the lower prevalence of serious illnesses seen in a general practice setting. Notably, these studies limited their outcome to serious bacterial infections only, and included children up to the age of 16 years in some cases.^11^^,^^12^

### Implications for research and practice

The conclusion reached is that the NICE traffic light system is not able to accurately detect or exclude serious illness in acutely unwell children presenting to general practice when the red category is used as a positive threshold. If this cohort’s traffic light classifications had been followed by GPs, a third of children (categorised into the red category) would have been urgently referred to hospital. Additionally, using the red category as a threshold for hospital referral would have missed 41.2% of children with a serious illness who were in the amber category, although NICE does recommend that clinicians should refer children in the amber category if indicated. Combining red and amber categories improved the sensitivity of the traffic light system, such that all seriously ill children were identified. This threshold would allow GPs to be confident sending children in the green category home, but at the cost of referring a substantial number of children to hospital; 94.3% of patients were categorised in the red or amber category. Moreover, GPs would only be able to confidently exclude serious illness in a minority (5.7%) of children classified in the green category.

The traffic light system was created to help GPs confidently assess unwell children, aiding their decisions about who to refer and who to send home by identifying those at risk of serious illness (thus prioritising sensitivity over specificity), but this study has shown that it is unable to accurately achieve this. This is an important finding in light of the current strain experienced by primary care services as a consequence of the COVID-19 pandemic. Secondary care can only function to assess and treat patients with serious illnesses provided there is effective functioning of primary care in serving the remainder.

Research is required to derive an updated tool for the assessment of acutely unwell children presenting to general practice. This assessment tool should correctly identify the most unwell children, while preventing unnecessary hospital referrals for children who are more likely to have a self-limiting illness. It should be derived and validated using data from UK general practice or primary care and must be suitable for use in a typical general practice consultation. This may require combining multiple datasets, because of the low prevalence of serious illness within this population. Future research could also assess whether ‘point-of-care’ markers of illness, such as C-reactive protein and procalcitonin, combined with clinical features in a single assessment tool could provide a more accurate indication of illness severity in children.

## Data

NHS Digital data re-use statement: Copyright © (2019), the Health and Social Care Information Centre. Re-used with the permission of the Health and Social Care Information Centre. All rights reserved. The patient datasets analysed in this study cannot be made available due to confidentiality and data-sharing agreements in place.
